# Contemporary management of anorectal fistula

**DOI:** 10.1016/j.sopen.2023.12.005

**Published:** 2024-01-02

**Authors:** Hyung Chan Kim, Vlad V. Simianu

**Affiliations:** a Department of Surgery, Virginia Mason Franciscan Health, Seattle, WA, United States of America; b Center for Digestive Health, Virginia Mason Franciscan Health, Seattle, WA, United States of America

**Keywords:** Anorectal fistula, crohn's disease, Seton, Fistulotomy

## Abstract

Anorectal fistula is a common, chronic condition, and is primarily managed surgically. Herein, we provide a contemporary review of the relevant etiology and anatomy anorectal fistula, treatment recommendations that summarize relevant outcomes and alternative considerations, in particular when to refer to a fistula expert.

## Introduction

Anorectal fistula is a relatively common, chronic connection between the anal canal and perianal skin [1]. It is considered primarily a “surgical disease,” as many patients will require some sort of operation or procedure to drain associated abscess or definitively treat the fistula. Therefore, an understanding of the relevant etiology and anatomy of an anorectal fistula, treatment options, and alternative considerations, in particular when to refer to a fistula expert, are critical in contemporary management. This review is not meant to exhaustively review the available evidence as is done by professional society guidelines [2] but rather proposes an approach to guide the majority of cases.

## Relevant etiology

The most commonly-accepted cause of anal fistula is that anal glands, which lie between the internal and external sphincters [3], get obstructed, develop an abscess, and the tract in which the abscess travels can become chronically inflamed and epithelialized. Anorectal fistula itself is not dangerous, but is extremely painful and affects the patient's quality of life. If the abscess is not adequately drained, it can lead to systemic infection. Patients may present with complaints of “hemorrhoids,” anal pain or itching, and depending on severity and chronicity [4], can also present with skin maceration, swelling, fever, or malodorous discharge.

Since anorectal fistulas vary in presentation as well as severity, similar to other anorectal diseases, the true prevalence of anorectal fistula is not exact, but estimated to be around 16–37 % of all patients presenting with anorectal abscesses [[5], [6], [7], [8]]. Although anorectal fistulae are more common in men than women, and in between 20 and 60 years of age, studies have not found significant differences in risk of recurrent anal abscess or fistula between gender, smoking status, HIV status, or alcohol use [5,9]. Additionally, more rare causes of anorectal fistulae include Crohn's disease, trauma, radiation, foreign body, infection, and malignancy [10]. These factors may be helpful at counseling patients about the relative complexity of their individual fistula, modifiable risk factors (if any), and considerations for surgical approach or referral.

**Fig. 1:**
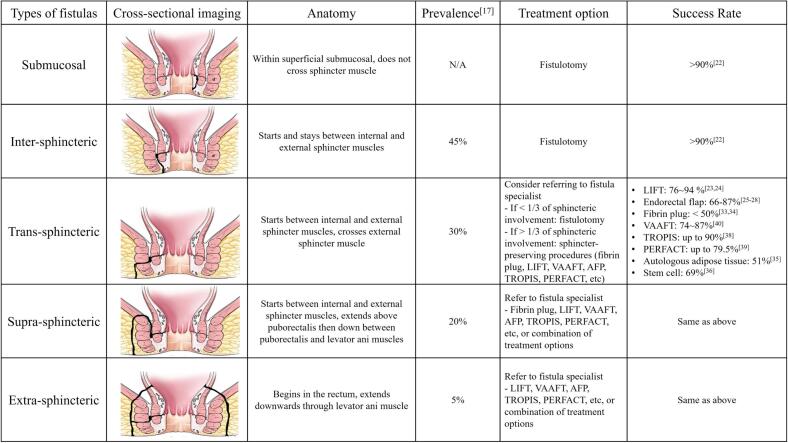
Types of anorectal fistulas, their anatomy, prevalence, treatment options and success rates. Cross-sectional images credits belong to The ASCRS Textbook of Colon and Rectal Surgery, 4th edition [4].

Any patient with concern for an anorectal fistula should first be evaluated with a detailed history and physical exam to determine the chronicity, symptoms, and other associated gastrointestinal, genitourinary, gynecological, and skin symptoms. The critical part is a physical examination of the anal canal to determine the fistula anatomy relative to the anal sphincters (Fig. 1). To define the anatomy of the fistula, the surgeon should do a thorough examination in the clinic or in the operating room using a combination of examination, digital rectal exam, and anoscopy with adjuncts like hydrogen peroxide or fistula probes to identify both internal and external openings. Most anorectal fistulae are simple and superficial, and this anatomy is typically confirmed in the operating room immediately before definitive treatment. Therefore, contemporary guidelines do not recommend routine imaging for most patients [2].

In cases of recurrent or occult fistula, or high suspicion for complexity (especially secondary to Crohn's disease), magnetic resonance imaging (MRI) or endoanal ultrasound (EAUS) can be used as adjuncts to help define the anatomy. MRI has been shown to correlate with operative/clinical findings in 82 % of anorectal fistulas [11]. Contrast may enhance the degree of inflammation, but there is also the downside of enhancing surrounding structures, adding confusion. Therefore, some radiologists do not recommend contrast enhancement, or if used, comparing the same sequence before and after contrast will give the most reliable image [12]. MRI may be able to identify findings not found on clinical exam or exam under anesthesia in up to 34 % of patients [13], leading to further “upstaging” of fistula grade and their potentially changing treatment. Studies have shown that EAUS is also a useful and valid imaging modality, demonstrating concordance with clinical and operative findings 73 to 90 % of the cases [14,15]. In addition, there is a potential additive role of combining modalities (e.g. clinical exam in OR with either preoperative MRI or ultrasound), something that can potentially help counsel patients on the relative ‘certainty’ of their fistula anatomy [2].

## Anatomic understanding is critical to deciding treatment

Although surgical treatment is mainstay of therapy, having a good understanding of the anatomy is vital [16]. Therefore, the commonly used classifications are all in relation to internal and external sphincter muscles: Park's classification, St James University Hospital classification, and Garg classification. Park's classification [17] was established in 1976 before advanced imaging such as MRI or EAUS were invented. St James University Classification in 2000 incorporated MRI, adding to the Park's classifications [18]. Then, Garg classification was established in 2017 [19], where the authors studied anorectal fistulas using MRI and operative findings to classify fistulas respective to their complexity and treatment. Herein, we will use 5 different anatomic configurations to propose treatment approaches in the contemporary era (Fig. 1).

Goal of surgical treatment for anal fistula is definitive treatment by closure, obliteration, or excision of the fistula tract, and the main priority is to avoid immediate fecal incontinence. While the fecal continence mechanism is complex, this loosely translates to a priority to preserve the external sphincter muscle across most approaches. The risk for post-operative anal sphincter dysfunction is typically when the anatomy is distorted, including recurrent or complex fistulas, previous anorectal surgeries, and female sex [20].

While treatment options available for any given situation may be many – seton placement, fistulotomy, fibrin glue injections, fistula plug, and advancement flaps – most surgeons have a few techniques they are comfortable with, and tailor these to the patient's situation and anatomy of the fistula.

## How-to: fistulotomy

For submucosal or simple intersphincteric fistulas, treatment is fistulotomy and healing by secondary intention is typically associated with low recurrence and complications [2,21,22]. In the operating room, a fistula probe should be passed through the fistula tract under anoscopy. The surgeon should palpate the distinct internal and external sphincter muscles – something that is often easier to do laterally along the anal canal compared to the anterior or posterior midline. The muscles should be traced to the fistula probe and confirmed to lie deep to the probe (submucosal fistula), or that only the internal sphincter (intersphincteric fistula) is involved. The fistula is then laid open with electrocautery to expose the entire, epithelialized tract. This tract can subsequently be debrided, and the edges can be marsupialized with absorbable suture. Primary fistulotomy is associated with high rate of symptom relief and fistula resolution rates (> 90 %) and risk of fecal incontinence is minimal in appropriately selected patients [2,5,21,22].

## How-to: seton placement

For deeper fistulas, involving the external sphincter muscle or having complexity like branching or extensive infection, the surgeon may opt for seton placement. A draining seton can provide symptomatic relief as well as decrease of inflammation, edema, or infection to allow for staged operative interventions. After identifying the fistula anatomy with the fistula probe as above, the extent of muscle involved or clinical situation (multiple branches, extensive infection, etc) are felt to be too involved for up-front treatment. The seton can typically be passed through the fistula using the fistula probe itself. Colored vessel loops are a common choice, but other options include permanent stitches or penrose drains. The seton is secured to itself using permanent suture. Setons management postoperatively can be individualized to the patient's situation – removed in clinic, removed as part of a staged fistula repair, or left indefinitely.

## Advanced treatments for anal fistula

A number of additional techniques have been described to treat complex anorectal fistulae and are summarized below:

• **LIFT (ligation of inter-sphincteric fistula tract)** involves dissection in the intersphincteric space until fistula tract is identified, then suture ligating and dividing the fistula tract [2]. A meta-analysis involving 1378 LIFT procedures reported a 76 % success rate, 14 % complication rate, and fecal incontinence rate of 1.4 % [23,24] . This operation may be more challenging in the posterior midline than in the lateral quadrants as the posterior midline anatomy is impacted by the anococcygeal ligaments.
• **Endorectal advancement flap** involves curetting the tract, closing with suture, and advancing segment of rectum to cover the internal opening [2]. Studies have demonstrated 66–87 % healing rate [[25], [26], [27], [28]], but given the possibility of internal anal sphincter muscles involved in the flap, there is a risk of some degree of incontinence [[29], [30], [31]]. In general it is accepted that a full-thickness flap may have better healing than partial thickness flap [32].
• **Fibrin plug** technique is an acellular collagen matrix injected into the internal fistula opening to obliterate the tract, combined with closure of the internal opening. It is generally considered prone to early failure, and ultimately healing rates of fibrin plug technique is 50 % or less [33,34], with poor long term healing [34].
• **Injection with freshly collected autologous adipose tissue** into anal fistulas [35] was studied as a treatment for anal fistulas in a prospective cohort study. It involved 77 patients, and after 6 months, 51 % of patients achieved fistula healing, 12 % decreased anal discomfort, and 4 % adverse events requiring surgical intervention (infection or bleeding). An important consideration is the protocol and equipment to derive the autologous adipose tissue, which is not widely available.
• **Stem cells in non-Crohn's anorectal fistula** [36] was studied in a prospective nonrandomized phase 1 clinical trial involving 20 patients. After the application, the patients were evaluated for a maximum of 1 year. No immediate adverse effects were reported in the first 24 h, and patients were discharged without complications. Complete closure was achieved in 69 % of patients, and 15 % of the patients developed perianal abscess. The results of the phase 3 randomized control trial in Crohn's patients [37] has not been officially published as of the writing of this manuscript.
• **TROPIS (trans-anal opening of intersphincteric tract)** [38] was a procedure first described in 2017, in which the fistula tract internal to external sphincter is opened into the anal canal, mostly reserved for complex fistulas. The initial study included high complex anorectal fistulas including supralevator and horseshoe fistula, and the healing rate was 90 %.
• **PERFACT (proximal superficial cauterization of mucosa at and around internal opening)** [39] involves curetting all tracts, cauterization at the internal opening to allow wound healing via secondary intention, and it is also reserved for highly complex anorectal fistulas. In the study published in 2015, 51 complex ano-fistula patients were included, the authors demonstrated 79.5 % success rate, and 20.5 % recurrence rate.
• **VAAFT (video-assisted anal fistula treatment)** was first described in 2011, where the authors used fistuloscope to identify the internal opening of the fistula and close it using a stapler, suture, or cutaneous-mucosal flap. The results are preliminary, as maximum follow up is 12 months, but healing rates range from 74 to 87 % at 1-year-period [40].

## Summary

Determining fistula anatomy is critical at deciding treatment and counseling the patient on likelihood of success or complication. Because many fistulae are subcutaneous or intersphincteric, they can be treated at the time of evaluation in the operating room with definitive fistulotomy. For complex fistulae, placement of a draining seton is a safe bridge to staged repair or referral to specialty expertise.

## Ethical approval

As this is an invited review paper, no formal ethical or human subjects review board was obtained.

## Funding sources

There are no relevant funding sources for this manuscript.

## CRediT authorship contribution statement

**Hyung Chan Kim:** Investigation, Methodology, Resources, Visualization, Writing – original draft. **Vlad V. Simianu:** Conceptualization, Methodology, Project administration, Resources, Supervision, Writing – review & editing.

## Declaration of competing interest

Vlad Simianu MD has received consulting fees from C-Sats, Inc., serves on the advisory board of BD Surgical, and has previously received educational travel support from Intuitive Surgical, Inc. The remaining authors have no conflicts of interest to disclose.
